# Integrated assessment of contrast-enhanced TCD and TTE for noninvasive detection of patent foramen ovale under resting and Valsalva conditions

**DOI:** 10.3389/fneur.2026.1751043

**Published:** 2026-03-13

**Authors:** Hui Yu, Yimin Wu, Yan Song, Pingyang Zhang, Haiyan Chen, Junli Wang

**Affiliations:** 1 Department of Ultrasound, WuHu Hospital, East China Normal University (The Second People's Hospital, WuHu), Wuhu, China; 2 Department of Echocardiography, Nanjing First Hospital, Nanjing Medical University, Nanjing, China; 3 Department of Echocardiography, Zhongshan Hospital, Fudan University, Shanghai, China

**Keywords:** decision curve analysis, diagnostic fusion model, patent foramen ovale, transcranial Doppler, transthoracic echocardiography, Valsalva maneuver

## Abstract

**Objective:**

To compare the diagnostic performance of contrast-enhanced transcranial Doppler (c-TCD) and contrast-enhanced transthoracic echocardiography (c-TTE) under resting and Valsalva conditions for detecting patent foramen ovale (PFO), and to evaluate the clinical utility of an integrated diagnostic model combining both modalities.

**Materials and methods:**

In this retrospective study, a total of 146 patients with suspected PFO underwent both c-TCD and c-TTE at rest and during the Valsalva maneuver. Among them, 40 patients also received contrast-enhanced transesophageal echocardiography (c-TEE), which served as the reference standard. Detection rates, shunt grading, and inter-modality agreement were analyzed. A logistic regression model integrating c-TCD and c-TTE findings was developed and assessed using receiver operating characteristic (ROC) curves and decision curve analysis (DCA).

**Results:**

The Valsalva maneuver significantly increased the detection rates of right-to-left shunt (c-TCD: from 41.4% to 76.7%; c-TTE: from 45.2% to 80.1%) and the proportion of Grade 3 shunts (*P* < 0.0001). At rest, c-TCD detected more high-grade shunts than c-TTE (*P* = 0.030), supporting its use as a sensitive initial screening tool. Inter-modality agreement improved markedly during Valsalva (weighted Kappa = 0.782). The combined model achieved 100% sensitivity and negative predictive value in the TEE subgroup, with an area under the ROC curve of 0.97 and no false negatives. DCA confirmed the superior net clinical benefit of the integrated approach across a broad range of decision thresholds.

**Conclusion:**

Valsalva maneuver significantly enhances the diagnostic yield of both c-TCD and c-TTE. While c-TCD may serve as an effective first-line screening tool, its combination with c-TTE ensures improved diagnostic accuracy and clinical decision-making value. The integrated model demonstrates strong potential for clinical implementation as a noninvasive, efficient strategy to reduce unnecessary c-TEE procedures.

## 1 Introduction

Patent foramen ovale (PFO) is a common congenital cardiac anomaly, present in approximately 25–30% of the general population and up to 50% in patients with cryptogenic stroke (1, 2). While frequently asymptomatic, PFO has been increasingly implicated as a conduit for paradoxical embolism and a potential risk factor for recurrent ischemic stroke, migraine with aura, decompression illness, and other embolic events (3). Accurate identification and functional characterization of PFO, particularly shunt severity and dynamic responsiveness are essential for risk stratification and therapeutic decision-making, especially in younger patients with embolic stroke of undetermined source (ESUS) (4).

Transesophageal echocardiography (TEE) is considered the gold standard for anatomical PFO confirmation due to its high spatial resolution and ability to directly visualize the interatrial septum and contrast flow (5). However, its semi-invasive nature, reliance on sedation, and limited patient tolerance hinder routine screening, especially in outpatient settings (6, 7). Consequently, contrast-enhanced transcranial Doppler (c-TCD) and contrast-enhanced transthoracic echocardiography (c-TTE) have become widely used noninvasive alternatives. c-TCD offers high sensitivity and real-time hemodynamic assessment by detecting microbubbles in the cerebral circulation, but lacks anatomical localization. c-TTE allows visualization of the cardiac origin of shunts but may be limited by acoustic window quality or small shunt volume (8–10).

Although both modalities have demonstrated clinical value, their performance varies significantly under different physiological conditions, especially between resting and Valsalva states (11). The Valsalva maneuver temporarily increases right atrial pressure, which can unmask latent shunts and enhance diagnostic yield (12, 13). However, comparative studies on the diagnostic accuracy, grading consistency, and inter-modality agreement of c-TCD and c-TTE under these conditions remain limited. Moreover, the potential benefit of integrating both modalities especially through statistical or machine learning-based fusion has not been comprehensively validated in a unified patient cohort using TEE as the reference (14–17).

In this study, we retrospectively analyzed 146 patients with suspected PFO who underwent both c-TCD and c-TTE under resting and Valsalva conditions. Among them, 40 patients underwent TEE for definitive diagnosis. We compared shunt detection rates, grading distributions, and inter-modality agreement under both conditions. In addition, we developed a logistic regression-based fusion model combining both modalities, and evaluated its diagnostic performance using ROC curve analysis, confusion matrices, and decision curve analysis (DCA). Our goal was to construct an optimized, noninvasive screening pathway that enhances sensitivity and clinical applicability while minimizing unnecessary TEE procedures.

## 2 Materials and methods

### 2.1 .1 Study population

This single-center retrospective study included 146 consecutive patients who underwent evaluation for suspected patent foramen ovale (PFO) at Wuhu Second People's Hospital between January 2022 and January 2025. The sample size was determined based on the number of consecutive eligible patients evaluated during the study period, without a formal power calculation. The primary symptoms prompting evaluation included migraine, cryptogenic stroke, and transient episodes of syncope. Among them, 50 were male and 96 were female, with an age range of 14–81 years (mean ± SD: 45.2 ± 15.4 years). All patients received both contrast-enhanced transcranial Doppler (c-TCD) and contrast-enhanced transthoracic echocardiography (c-TTE). Of these, 40 patients additionally underwent transesophageal echocardiography (TEE) within 1 week, which served as the reference standard. Inclusion criteria were: (1) presence of symptoms suggestive of right-to-left shunt such as migraine, cryptogenic stroke, transient ischemic attack, or unexplained syncope; (2) complete c-TCD and c-TTE data. Exclusion criteria included: (1) inadequate bilateral temporal acoustic windows preventing c-TCD assessment; (2) inability to perform an effective Valsalva maneuver or to complete the TEE procedure; (3) known atrial septal defect or other structural heart disease. This study was approved by the institutional ethics committee (2022-JS-068). Given its retrospective design, the requirement for written informed consent was waived.

### 2.2 Examination protocols

#### 2.2.1 Contrast-enhanced transcranial Doppler (c-TCD)

All c-TCD examinations were performed using the Delica 9D Doppler ultrasound system, equipped with a 1.6 MHz probe. The patients were positioned in the supine position, with an 18G cannula inserted into the left cubital vein. The right middle cerebral artery (MCA) was monitored through the superior temporal bone window. The first injection was performed in the resting state, followed by a second injection after the patient performed the classic Valsalva maneuver. The Valsalva maneuver involved normal or deep inhalation, followed by glottis closure and forceful exhalation, maintaining the maneuver for at least 5 s to ensure that microbubbles filled the right atrium. Microbubble signals were recorded for 25 s post-injection. The preparation of the agitated saline solution involved mixing 8 mL of saline, 1 mL of the patient's blood, and 1 mL of room air, which was agitated at least 20 times between two 10 mL syringes to prepare a good contrast agent. The agitated saline solution was injected 5 s before the start of the Valsalva maneuver and maintained for at least 10 s. Doppler signals from MCA were recorded, with three repetitions of the Valsalva maneuver, each separated by a 5 min interval, to avoid misinterpretation. The final result was based on the most intense high-intensity transient signals (HITS) recorded during MCA monitoring. Shunt severity was assessed using a semi-quantitative visual grading approach based on microbubble signals, rather than exact numerical counting. Shunt Classification: The modified 4-level classification (unilateral middle cerebral artery) was used as follows: Grade 0: No microbubbles, no shunt; Grade 1: 1–10 microbubbles, minimal right-to-left shunt (RLS); Grade 2: 11–25 microbubbles, moderate RLS; Grade 3: >25 microbubbles, large RLS, or “curtain-like” pattern (18).

#### 2.2.2 Contrast-enhanced transthoracic echocardiography (c-TTE)

The c-TTE examinations were performed using the GE Vivid E9 or Vivid E95 ultrasound systems, equipped with the S5-1 probe (1–5 MHz). Routine c-TTE was performed to exclude other causes of cardiac shunt before the injection of the contrast mixture. During both the resting state and the Valsalva maneuver, the apical four-chamber view was used to continuously record the number of microbubbles. To ensure the adequacy of the Valsalva maneuver, patients were routinely trained and practiced the maneuver before the examination. The Valsalva maneuver was maintained for at least 5 s to ensure the right atrium was filled with microbubbles. The agitated saline solution was injected a few seconds before the Valsalva maneuver and maintained until the right atrium was filled with the contrast agent. If the test result was positive, two additional injections were performed to assess reproducibility. The final result was based on the maximal visual microbubble burden observed in the left ventricle. Shunt severity was evaluated using a semi-quantitative grading system based on visual assessment of microbubble appearance rather than exact counting. The shunt severity classification was as follows: Grade 0: No microbubbles detected; Grade I: Mild (1–9 microbubbles); Grade II: Moderate (10–30 microbubbles); Grade III: Severe (>30 microbubbles or the left ventricle nearly filled with microbubbles) (19).

#### 2.2.3 Contrast-enhanced transesophageal echocardiography (c-TEE)

**Figure 1:**
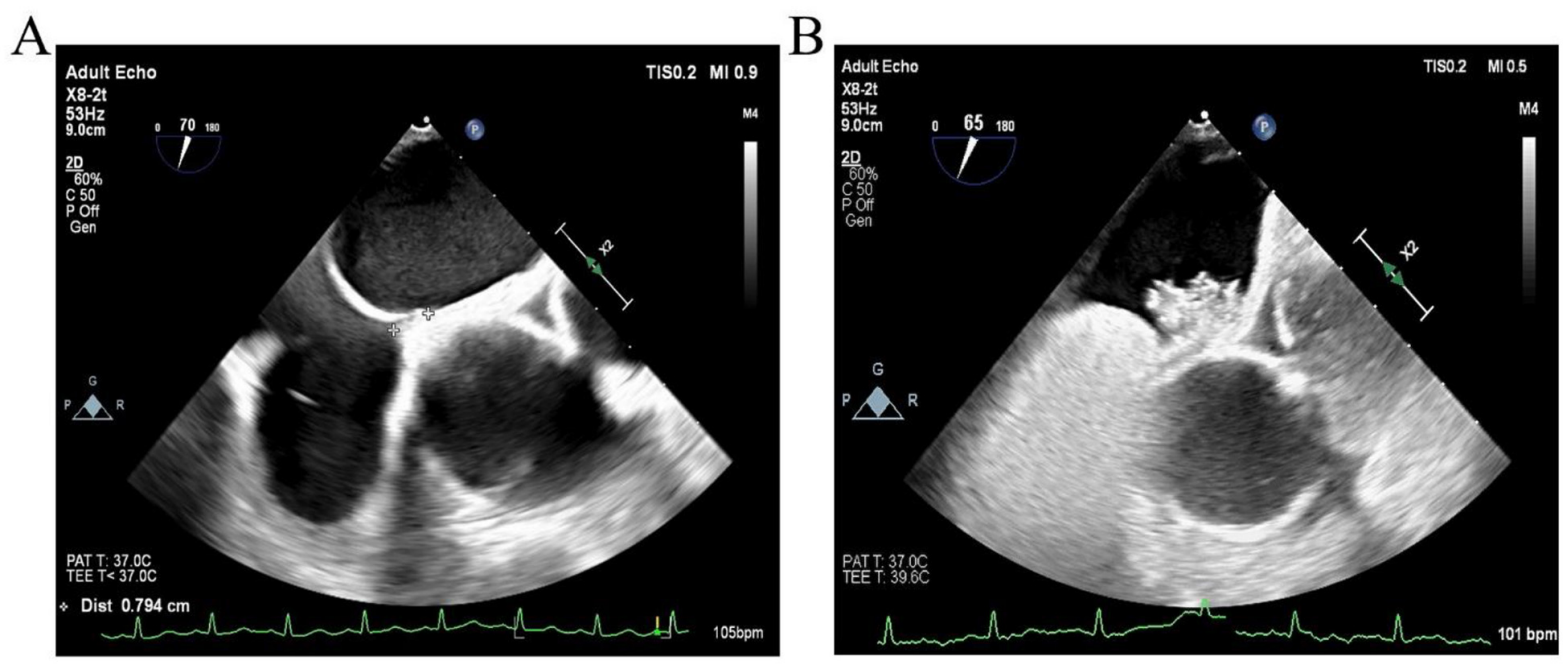
Transesophageal echocardiography (c-TEE) findings. **(A)** Measurement of the PFO tunnel length during Valsalva maneuver. **(B)** Real-time visualization of microbubble passage from the right to left atrium through the foramen ovale, confirming the presence of a right-to-left shunt (RLS).

The c-TEE examinations were performed using the Philips Epiq 7c system equipped with an X7-2t multiplane probe (5–7 MHz). All patients fasted for 12 h before the procedure and were placed in the left lateral decubitus position. A local anesthesia was administered using 1 tube of Dyclonine gel for oropharyngeal anesthesia. Following probe insertion, a comprehensive two-dimensional, color Doppler, and spectral Doppler transesophageal echocardiographic examination was performed. The atrial septum and surrounding structures were visualized in the mid-esophageal bicaval view with an angle of 30° to 110° (Figure 1). During the Valsalva maneuver, the ultrasound technologist asked the patients to expand their abdomen and simultaneously applied hand pressure to increase intra-abdominal pressure. The agitated saline was injected a few seconds after the start of the Valsalva maneuver. Continuous recording was performed during both basal conditions and the Valsalva maneuver. A PFO was considered present if microbubbles were observed in real time passing from the right atrium into the left atrium, either at rest or during the Valsalva maneuver, with particular emphasis on Valsalva-provoked shunting.

### 2.3 Statistical analysis

All statistical analyses were conducted using SPSS version 27.0 (IBM Corp., Armonk, NY, USA), GraphPad Prism version 9.0 (GraphPad Software Inc.), and R version 4.2.1 (R Foundation for Statistical Computing, Vienna, Austria). Continuous variables were presented as mean ± standard deviation (SD) or median with interquartile range [IQR], and compared using the Student's *t*-test or Wilcoxon rank-sum test, depending on normality. Categorical variables were summarized as frequencies and percentages, and compared using the chi-square test or Fisher's exact test as appropriate. Paired categorical variables, such as detection rates and shunt grades between modalities, were analyzed using McNemar's test or Wilcoxon signed-rank test. To evaluate the diagnostic performance of each modality (c-TCD, c-TTE, and their combination) using TEE as the reference standard, sensitivity, specificity, positive predictive value (PPV), negative predictive value (NPV), and Youden index were calculated. Receiver operating characteristic (ROC) curves were plotted, and the area under the curve (AUC) was used to compare discriminative performance. Binary logistic regression analysis was performed to assess the association between shunt grade (as an ordinal predictor) and the likelihood of PFO confirmed by TEE. In addition, a combined diagnostic model incorporating c-TCD and c-TTE results was constructed using logistic regression, and its performance was compared with individual modalities. Decision curve analysis (DCA) was performed using the dcurves package in R to estimate the net clinical benefit of each diagnostic approach across a range of threshold probabilities. A two-tailed *P*-value < 0.05 was considered statistically significant.

## 3 Results

### 3.1 Baseline characteristics

Of the 156 patients initially evaluated for suspected PFO during the study period, 10 were excluded due to inadequate temporal acoustic windows (*n* = 4), inability to complete Valsalva maneuver (*n* = 4), or presence of known atrial septal defect or other structural heart disease (*n* = 3). A total of 146 patients were included, with a mean age of 45.2 ± 15.4 years (range: 14–81 years), and 96 (65.8%) were female. Clinical indications included migraine (*n* = 101), transient ischemic attack (*n* = 12), and cryptogenic stroke (*n* = 33). Table 1 summarizes demographic and clinical characteristics.

**Table 1:** Baseline characteristics of the study population.

**Characteristic**	**Values**
Age (years)	45.2 ± 15.4
**Sex**, ***n*** **(%)**	
Male	50 (34.2%)
Female	96 (65.8%)
**Symptom**, ***n*** **(%)**	
Migraine	101 (69.2%)
Transient ischemic attack	12 (8.2%)
Cryptogenic stroke	33 (22.6%)
c-TCD positive (rest), *n* (%)	60 (41.1%)
c-TCD positive (valsalva), *n* (%)	112 (76.7%)
c-TTE positive (rest), *n* (%)	66 (45.2%)
c-TTE positive (valsalva), *n* (%)	117 (80.1%)
Underwent c-TEE, *n* (%)	40 (27.4%)
c-TEE positive (among tested), *n* (%)	34 (85.0%)
c-TEE negative (among tested), *n* (%)	6 (15.0%)

c-TCD, Contrast transcranial Doppler; c-TTE, Contrast transthoracic echocardiography; c-TEE, Contrast transesophageal echocardiography.

### 3.2 Detection rates and shunt grading comparison

**Figure 2:**
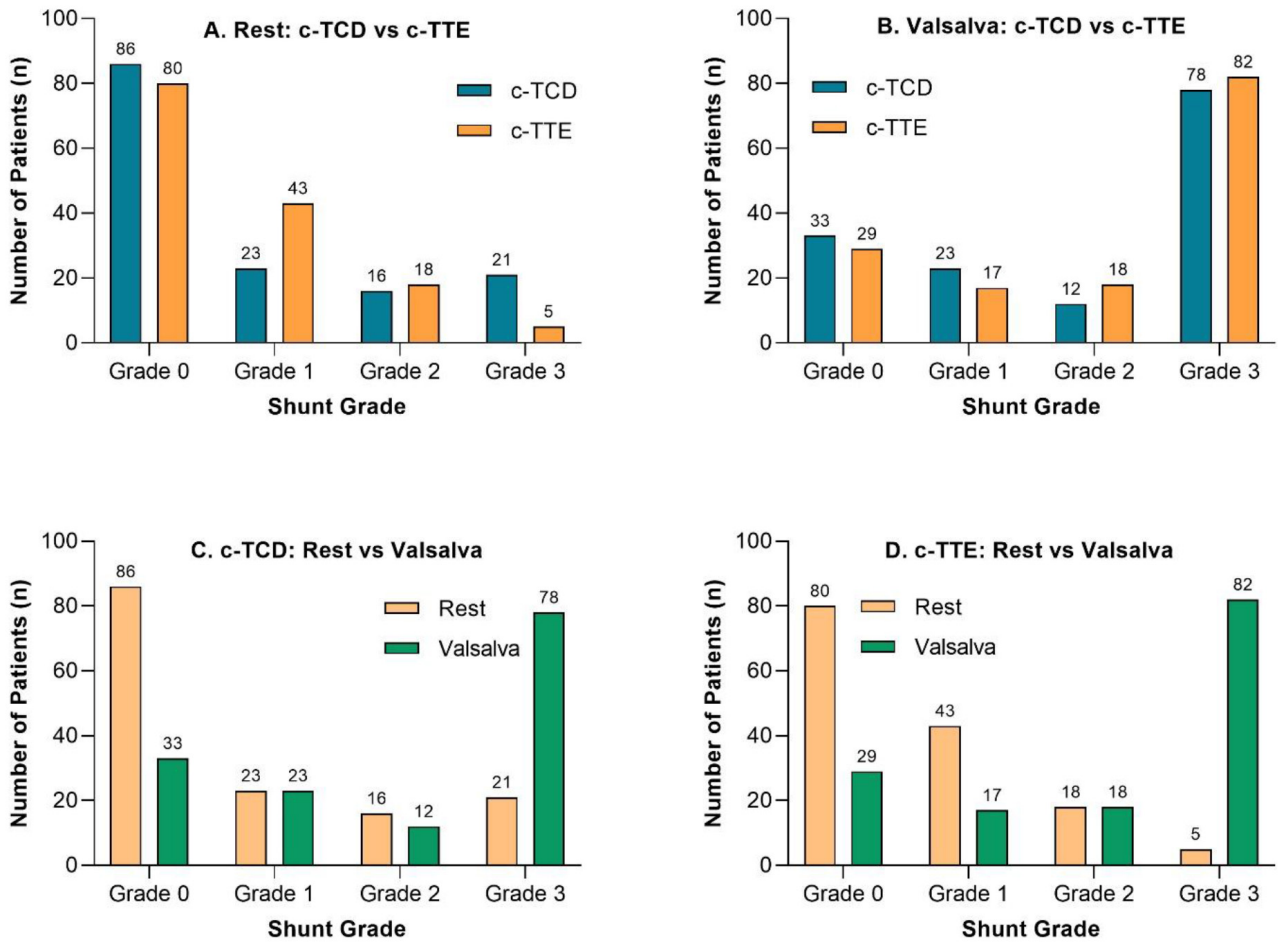
Comparison of shunt grades detected by c-TCD and c-TTE under resting and Valsalva conditions. **(A)** Distribution of shunt grades (0–3) detected by c-TCD and c-TTE at rest. **(B)** Distribution of shunt grades detected by c-TCD and c-TTE during the Valsalva maneuver. **(C)** Changes in c-TCD-detected shunt grades from rest to Valsalva. **(D)** Changes in c-TTE-detected shunt grades from rest to Valsalva. Valsalva maneuver significantly increased the detection of Grade 3 shunts for both modalities.

As shown in Table 2A, there was no statistically significant difference between c-TCD and c-TTE in detecting right-to-left shunt (RLS) during either the resting state (*P* = 0.392) or the Valsalva maneuver (*P* = 0.267), according to McNemar's test. However, compared to the resting state, the detection rates increased substantially for both modalities during the Valsalva maneuver, with c-TCD rising from 41.1% to 76.7% and c-TTE from 45.2% to 80.1%. Table 2B further presents the semiquantitative grading distributions (Grade 0–3) of RLS, showing that during rest, c-TCD demonstrated significantly higher shunt grades than c-TTE (*P* = 0.030), while this difference was no longer significant under Valsalva (*P* = 0.072). Within each modality, the distribution of shunt grades shifted significantly during Valsalva (both *P* < 0.0001, Wilcoxon signed-rank test), with Grade 3 detection increasing markedly-from 21 to 78 in c-TCD, and from 5 to 82 in c-TTE-indicating that Valsalva substantially enhances the identification of high-grade shunts. As shown in Table 2C, inter-modality agreement also improved under Valsalva: based on RLS detection (positive/negative), Kappa values increased from 0.526 (rest) to 0.737 (Valsalva), and based on shunt grade (0–3), weighted Kappa values rose from 0.599 to 0.782 (all *P* < 0.001), suggesting that the Valsalva maneuver not only improves detection sensitivity but also enhances grading consistency between modalities. These findings are visually illustrated in Figure 2, which shows that both c-TCD and c-TTE detected a substantially higher number of Grade 3 shunts during the Valsalva maneuver compared to the resting state.

**Table 2:** Detection rates and shunt grading comparison between c-TCD and c-TTE.

**A. Detection rates at rest and during Valsalva maneuver**				
**Detection conditions**	**Positive**, ***n*** **(%)**		**Negative**, ***n*** **(%)**	
**Rest**				
c-TCD	60 (41.4%)		86 (58.9%)	
c-TTE	66 (45.2%)		80 (54.8%)	
**Valsalva**				
c-TCD	112 (76.7%)		34 (23.3%)	
c-TTE	117 (80.1%)		29 (19.9%)	
McNemar test (Rest): *P* = 0.392; McNemar test (Valsalva): *P* = 0.267.				
**B. Shunt grading distribution and comparison**				
**(0–3 scale)**
**Shunt Grade**	**c-TCD (Rest)**	**c-TCD (Valsalva)**	**c-TTE (Rest)**	**c-TTE (Valsalva)**
Grade 0	86	33	80	29
Grade 1	23	23	43	17
Grade 2	16	12	18	18
Grade 3	21	78	5	82
Wilcoxon signed-rank test: Rest (c-TCD vs. c-TTE): P = 0.030; Valsalva
(c-TCD vs. c-TTE): *P* = 0.072; c-TCD: *P* < 0.0001; c-TTE: *P* < 0.0001.
**C. Agreement between c-TCD and c-TTE**				
**Metric**			**Rest**	**Valsalva**
RLS detection (positive/negative)—Kappa coefficient			0.526	0.737
*P*-value (RLS detection)			< 0.001	< 0.001
Shunt grade (0–3)—weighted Kappa			0.599	0.782
*P*-value (Shunt grade)			< 0.001	< 0.001
c-TCD, Contrast transcranial Doppler; c-TTE, Contrast transthoracic				
echocardiography.				

### 3.3 Diagnostic performance compared to TEE

**Figure 3:**
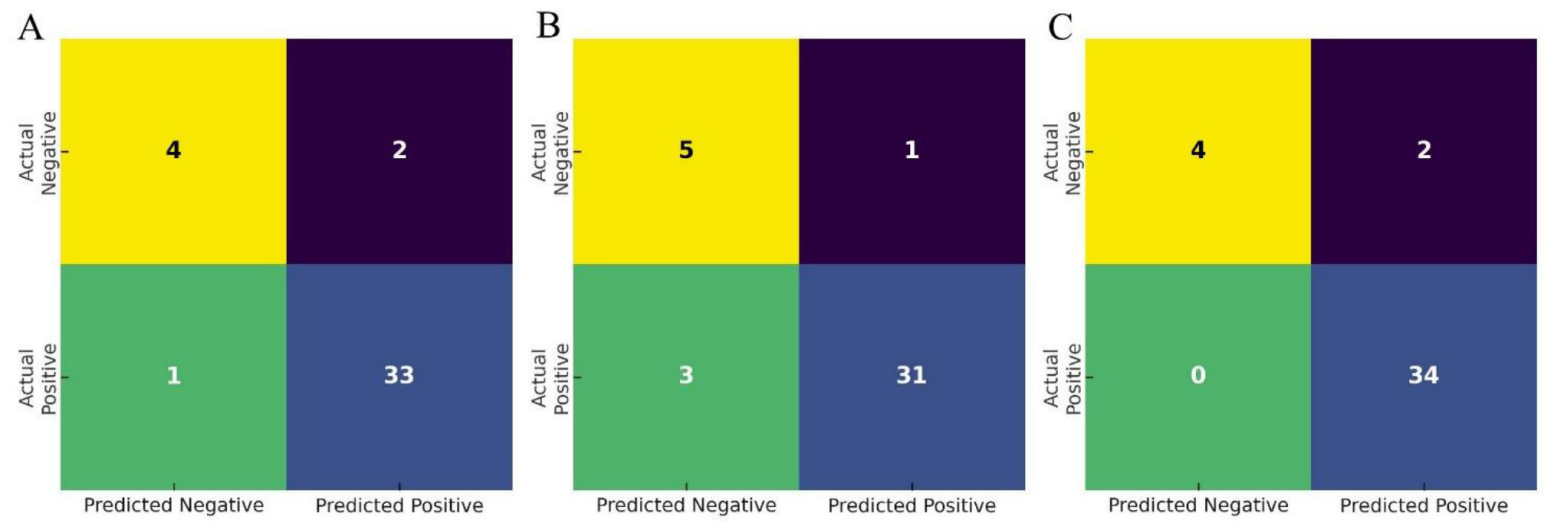
Confusion matrices illustrating the classification performance of each diagnostic approach using TEE as the reference standard. **(A)** c-TCD model. **(B)** c-TTE model. **(C)** Combined logistic regression model. The combined model achieved perfect sensitivity and negative predictive value (NPV), eliminating false negatives.

**Figure 4:**
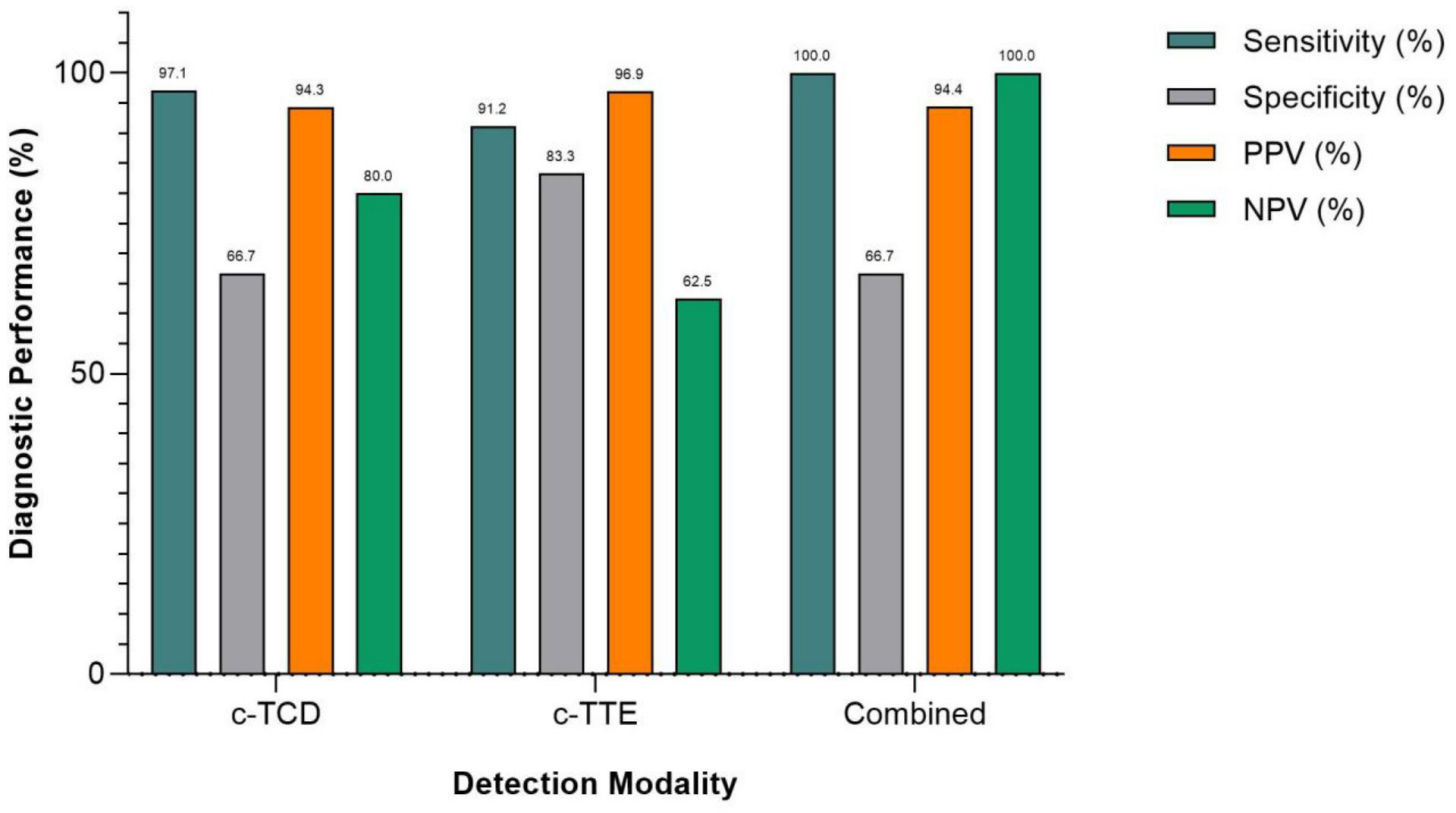
Bar chart comparing key diagnostic performance metrics of the three modalities: contrast-enhanced transcranial Doppler (c-TCD), contrast-enhanced transthoracic echocardiography (c-TTE), and the combined model. Metrics include sensitivity, specificity, positive predictive value (PPV), and negative predictive value (NPV). The combined model yielded 100% sensitivity and NPV, underscoring its value as a safe screening tool for ruling out PFO.

Among the 40 patients who completed all three examinations (c-TCD, c-TTE, and c-TEE), the diagnostic performance of each modality was assessed using c-TEE as the reference standard. As shown in Table 3, c-TCD exhibited the highest sensitivity (97.1%) but relatively low specificity (66.7%), indicating strong capability to identify PFO cases but a higher false-positive rate. In contrast, c-TTE showed higher specificity (83.3%) with a slightly lower sensitivity (91.2%), representing a more balanced diagnostic profile. The combined diagnostic model, integrating both c-TCD and c-TTE results via logistic regression, achieved perfect sensitivity (100.0%) and negative predictive value (NPV, 100.0%), meaning no false negatives were observed, while its specificity remained the same as c-TCD (66.7%). This finding suggests that the combined model is particularly valuable for screening purposes, where ruling out disease with high confidence is critical. Figures 3A–C presents the confusion matrices for c-TCD, c-TTE, and the combined model. While both individual methods produced false positives or false negatives to varying degrees, the combined model successfully eliminated all false negatives, underscoring its screening safety. Figure 4 compares key diagnostic indices across the three approaches. The combined model outperformed c-TTE and c-TCD in sensitivity and NPV, and matched c-TTE in PPV, though it did not improve specificity. These results indicate that the combined approach maximizes true positive identification while minimizing the risk of missed PFO cases, making it a robust and clinically safe initial screening strategy.

**Table 3:** Diagnostic performance of c-TCD, c-TTE, and combined testing compared with c-TEE (*n* = 40).

**Modality**	**Sensitivity (%)**	**Specificity (%)**	**PPV (%)**	**NPV (%)**	**Youden index**
c-TCD	97.1	66.7	94.3	80.0	0.637
c-TTE	91.2	83.3	96.9	62.5	0.745
Combined	100.0	66.7	94.4	100.0	0.667

c-TCD, Contrast transcranial Doppler; c-TTE, Contrast transthoracic echocardiography; c-TEE, Contrast transesophageal echocardiography; PPV, positive predictive value; NPV, negative predictive value.

### 3.4 Comprehensive model evaluation: AUC, OR, clinical utility, and individualized prediction

**Figure 5:**
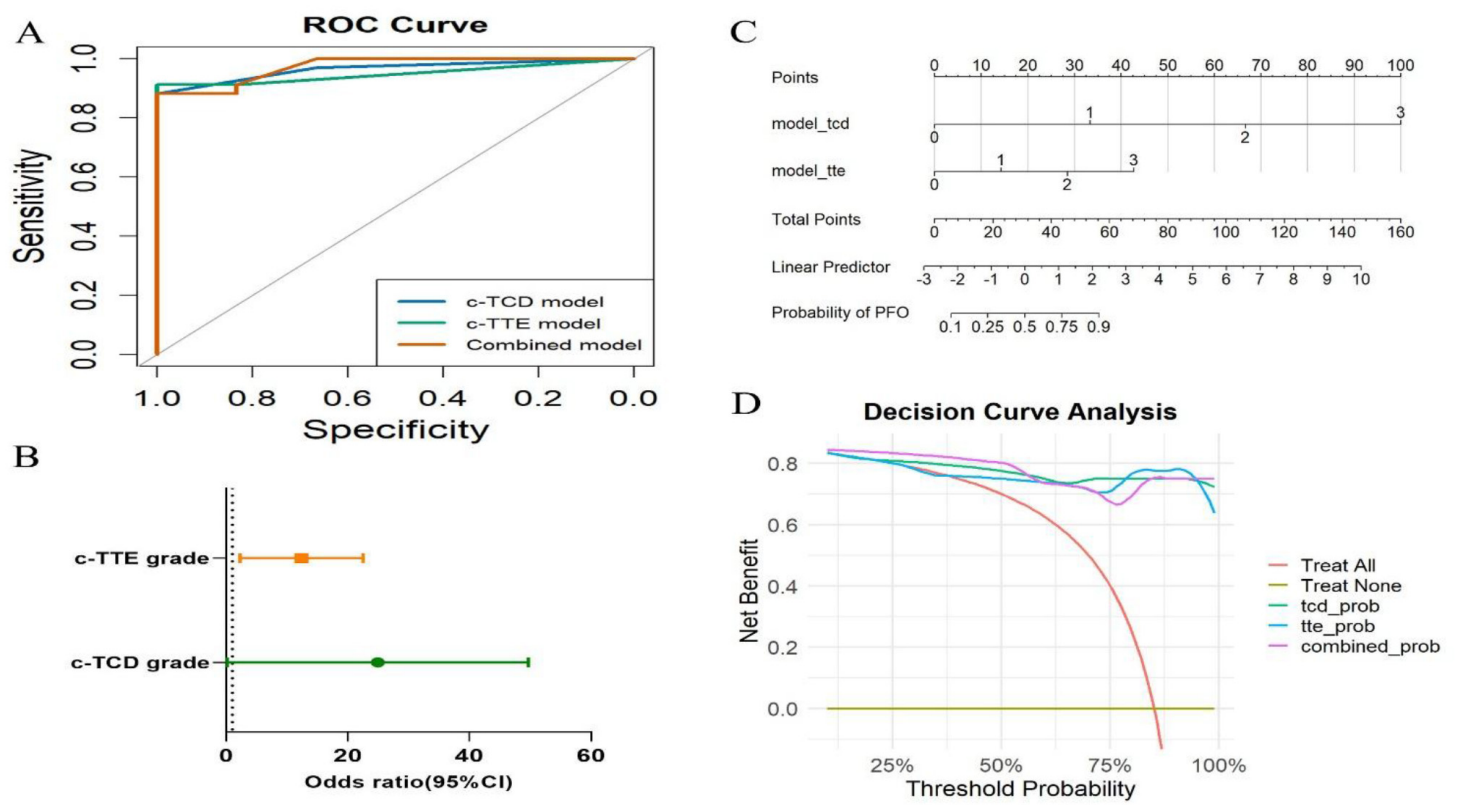
Comprehensive evaluation of model performance and clinical utility. **(A)** Receiver operating characteristic (ROC) curves of the three models for predicting PFO. **(B)** Forest plot showing odds ratios (ORs) for shunt grades detected by c-TCD and c-TTE, with increasing grades associated with higher odds of confirmed PFO. **(C)** Nomogram integrating c-TCD and c-TTE grades for individualized PFO probability estimation. **(D)** Decision curve analysis (DCA) showing that the combined model provides the highest net clinical benefit across a threshold probability range of 0.1–0.6.

To comprehensively evaluate the diagnostic performance and clinical utility of the three models, we conducted a series of quantitative assessments, as summarized in Figures 5A–D. Figure 5A shows the receiver operating characteristic (ROC) curves. All three models exhibited excellent discrimination: c-TCD achieved an AUC of 0.97 (95% CI: 0.92–1.00), c-TTE had an AUC of 0.95 (95% CI: 0.89–1.00), and the combined model reached an AUC of 0.97 (95% CI: 0.93–1.00). Although the combined model's AUC was comparable to that of c-TCD, it achieved 100% sensitivity and negative predictive value (NPV) in this cohort, with no false negatives observed, indicating superior screening safety and utility. To assess the clinical relevance of semiquantitative shunt grading, we performed logistic regression analysis using c-TCD and c-TTE grades as independent predictors of TEE-confirmed PFO. As illustrated in Figure 5B, higher shunt grades were significantly associated with increased odds of confirmed PFO, and the odds ratios increased progressively from Grade 1 to Grade 3 in both modalities. To facilitate individualized risk estimation, we constructed a nomogram based on the fusion logistic regression model integrating both c-TCD and c-TTE grades (Figure 5C). Each combination of input variables corresponds to a total point score, which maps to an estimated probability of TEE-confirmed PFO. This nomogram provides a quantitative, patient-specific probability output based on combined c-TCD and c-TTE findings, facilitating individualized risk estimation and supporting triage-oriented interpretation of noninvasive screening results. Finally, Figure 5D presents the results of decision curve analysis (DCA). Across a wide range of threshold probabilities (0.1–0.6), the combined model consistently demonstrated the greatest net benefit compared to either modality alone, highlighting its potential utility in real-world, patient-centered decision-making.

## 4 Discussion

This study presents a comprehensive, comparative analysis of c-TCD and c-TTE for PFO detection under both resting and Valsalva states, and further proposes a fusion diagnostic model validated against TEE. Five key findings emerged: (1) Valsalva maneuver significantly increased the detection rate and grading of right-to-left shunt (RLS) for both modalities, especially for Grade 3 shunts; (2) c-TCD demonstrated higher sensitivity than c-TTE under resting conditions, with statistically significant differences in shunt grading distribution; (3) the agreement between c-TCD and c-TTE improved substantially under Valsalva, as shown by weighted Kappa values; (4) the fusion model achieved perfect sensitivity and negative predictive value (NPV) with TEE validation, outperforming individual modalities; (5) decision curve analysis (DCA) revealed that the combined model provided the greatest net clinical benefit across a range of threshold probabilities. These findings provide important evidence supporting a multimodal, physiology-sensitive, and decision-oriented pathway for noninvasive PFO screening.

Our results highlight the pivotal role of the Valsalva maneuver in RLS detection and quantification. During Valsalva, c-TCD and c-TTE demonstrated marked increases in Grade 3 shunt detection-from 21 to 78 and 5 to 82 cases, respectively indicating improved hemodynamic provocation. Notably, the weighted Kappa for shunt grading between c-TCD and c-TTE rose from 0.599 (moderate) at rest to 0.782 (substantial) during Valsalva. These findings corroborate existing evidence that physiologic activation enhances right-to-left pressure gradients and standardizes inter-modality grading (11, 20). In contrast, TEE often suffers from reduced Valsalva effectiveness due to patient sedation or discomfort, which may impair diagnostic accuracy even under optimal image acquisition (21, 22). Collectively, our data suggest that careful Valsalva monitoring in TCD/TTE protocols may offer more consistent shunt detection in ambulatory settings.

Beyond Valsalva-provoked shunting, previous studies (23) have shown that RLS through a patent foramen ovale may be posture dependent in a subset of patients, particularly under spontaneous breathing conditions. Importantly, postural variations in shunt magnitude appear to be most pronounced in the absence of the Valsalva maneuver and may become attenuated once Valsalva induces maximal functional opening of the interatrial septum. In the present study, we focused on standardized resting and Valsalva conditions, with particular emphasis on Valsalva-provoked shunting, which yielded the highest detection rates, improved grading consistency, and optimal fusion model performance. Although positional testing was not systematically performed, contrast-enhanced transcranial Doppler remains uniquely suited for evaluating posture-dependent shunting under daily physiological conditions, and this aspect warrants further investigation in future studies.

Consistent with prior meta-analyses, our findings reaffirm that c-TCD offers the highest sensitivity (97.1%) while c-TTE provides stronger specificity (83.3%) (11). Importantly, our logistic regression-based fusion model achieved 100% sensitivity and negative predictive value (NPV), eliminating false negatives within the TEE-validated subgroup. While c-TCD may overcall RLS due to its inability to distinguish cardiac from pulmonary shunts (24), c-TTE complements this by providing anatomical confirmation of interatrial septal passage. Several studies have advocated a “TCD-first, TTE-refinement” paradigm (25, 26), but our integrated approach formalizes this logic into a quantifiable probability output, increasing its interpretability and clinical utility. In this context, the nomogram provides an individualized probability of TEE-confirmed PFO based on combined c-TCD and c-TTE findings. Clinically, a higher predicted probability may support referral for confirmatory TEE, whereas a lower predicted probability may help defer invasive testing in selected screening scenarios.

To bridge the gap between statistical performance and clinical decision-making, we incorporated decision curve analysis (DCA) alongside ROC and logistic metrics. The fusion model consistently demonstrated the highest net benefit within a clinically relevant threshold probability range (0.1–0.6). This aligns with the increasing application of decision curve analysis (DCA) in radiology and cardiovascular outcome research, where DCA provides an intuitive framework to evaluate whether a patient should undergo confirmatory TEE or proceed directly to clinical intervention based on individualized risk modeling (27, 28). The strength of DCA lies in its integration of outcome probabilities with real-world utility, especially in ambiguous cases where PFO closure decisions hinge on diagnostic certainty. In line with recent consensus statements from European stroke and cardiology societies, our findings support a multimodal algorithm anchored in initial noninvasive screening (29). To further demonstrate the model's practical value in clinical scenarios, we analyzed two representative cases with divergent c-TCD and c-TTE findings. In one case, a 46-year-old female patient exhibited Grade 0 shunt on c-TCD under both resting and Valsalva conditions, but showed Grade 3 shunt on c-TTE. While the isolated c-TCD result might have led to underestimation, the fusion model correctly classified her as high-risk. TEE subsequently confirmed a significant PFO, illustrating how the model may reduce missed diagnoses and ensure appropriate referral. Conversely, in another case, a 35-year-old female with a Grade 3 shunt on c-TCD and only mild to moderate shunt (Grade 1 at rest, Grade 2 during Valsalva) on c-TTE received a moderate-risk score from the fusion model. TEE confirmed a small, hemodynamically less significant PFO. This case underscores the model's ability to integrate anatomical and hemodynamic inputs, thereby avoiding overtreatment or unnecessary invasive testing. These illustrative cases highlight the fusion model's potential to improve risk stratification, reconcile inter-modality discrepancies, and optimize individualized clinical decision-making in real-world settings.

This study offers several methodological and translational strengths: (1) dual-condition comparison of RLS detection using two complementary modalities; (2) rigorous application of shunt grading systems and inter-modality agreement metrics; (3) development of an interpretable logistic regression-based fusion model; and (4) incorporation of decision curve analysis (DCA) to evaluate clinical utility in real-world settings. Nevertheless, several limitations should be acknowledged. Nevertheless, several limitations should be acknowledged. Transesophageal echocardiography (TEE) was performed in a subset of patients based on clinical judgment rather than uniformly across the entire cohort, reflecting real-world diagnostic workflows; consequently, the TEE-validated subgroup was of moderate size. In addition, the study population included patients with heterogeneous clinical presentations, such as migraine and stroke/TIA, reflecting a screening-oriented cohort rather than a closure-focused population; subgroup-specific diagnostic performance was therefore not separately analyzed and warrants further investigation in future studies. The effectiveness of the Valsalva maneuver may also vary depending on operator coaching and patient cooperation, which could influence shunt detection and grading. Furthermore, the fusion diagnostic model was constructed using classical statistical methods rather than machine learning algorithms. Building on prior studies that have successfully applied artificial intelligence to TCD signal interpretation (14, 17), future research should aim to integrate such algorithms into multimodal diagnostic frameworks and validate their performance in large, multicenter cohorts, ideally through prospective study designs and external validation in independent populations, ultimately supporting rapid, standardized, and patient-centered PFO screening.

This study demonstrates that Valsalva maneuver significantly enhances the diagnostic performance and agreement of c-TCD and c-TTE in detecting PFO. A fusion model combining both modalities achieved excellent sensitivity and clinical utility, outperforming either method alone. These findings support a noninvasive, multimodal screening pathway for PFO and align with emerging AI-assisted diagnostic strategies.

## Data Availability

The datasets presented in this article are not readily available because the datasets used in this study are based on retrospective clinical records from Wuhu Second People's Hospital and contain potentially sensitive patient information. Due to institutional and ethical restrictions, these data cannot be publicly shared. Anonymized summary data that support the findings of this study are available from the corresponding author upon reasonable request. Requests to access the datasets should be directed to Junli Wang, wjl980134@163.com.
